# Human and Mouse Hematopoietic Stem Cells Are a Depot for Dormant *Mycobacterium tuberculosis*

**DOI:** 10.1371/journal.pone.0169119

**Published:** 2017-01-03

**Authors:** Julia Tornack, Stephen T. Reece, Wolfgang M. Bauer, Alexis Vogelzang, Silke Bandermann, Ulrike Zedler, Georg Stingl, Stefan H. E. Kaufmann, Fritz Melchers

**Affiliations:** 1 Senior Group on Lymphocyte Development, Max Planck Institute for Infection Biology, Berlin, Germany; 2 Department of Immunology, Max Planck Institute for Infection Biology, Berlin, Germany; 3 Division of Immunology, Allergy and Infectious Diseases, Department of Dermatology, Medical University of Vienna, Vienna, Austria; Rutgers Biomedical and Health Sciences, UNITED STATES

## Abstract

An estimated third of the world’s population is latently infected with *Mycobacterium tuberculosis* (*Mtb*), with no clinical signs of tuberculosis (TB), but lifelong risk of reactivation to active disease. The niches of persisting bacteria during latent TB infection remain unclear. We detect *Mtb* DNA in peripheral blood selectively in long-term repopulating pluripotent hematopoietic stem cells (LT-pHSCs) as well as in mesenchymal stem cells from latently infected human donors. In mice infected with low numbers of *Mtb*, that do not develop active disease we, again, find LT-pHSCs selectively infected with *Mtb*. In human and mouse LT-pHSCs *Mtb* are stressed or dormant, non-replicating bacteria. Intratracheal injection of *Mtb*-infected human and mouse LT-pHSCs into immune-deficient mice resuscitates *Mtb* to replicating bacteria within the lung, accompanied by signs of active infection. We conclude that LT-pHSCs, together with MSCs of *Mtb*-infected humans and mice serve as a hitherto unappreciated quiescent cellular depot for *Mtb* during latent TB infection.

## Introduction

Tuberculosis (TB) is a major infectious disease in humans, caused by *Mycobacterium tuberculosis* (*Mtb*), with 9.6 million active cases reported in 2014. A much larger part of the world’s population is, in addition, latently TB-infected (LTBI), with no clinical signs of disease, but lifelong risk of reactivation. *Mtb* can enter dormancy, thereby persisting in the human host despite a strong immune response [1, 2]. How, where, and under what circumstances *Mtb* is retained in the host during LTBI, and how it could undergo resuscitation and cause active TB, remain important but unresolved questions [3–5]. We test the hypothesis that in LTBI *Mtb* bacteria acquire a non-replicating state inside resting, long-term repopulating pluripotent hematopoietic stem cells (LT-pHSCs) [6]. LT-pHSCs tolerate hypoxia [7, 8], lack tuberculocidal activity mediated by endogenous reactive oxygen and nitrogen species [9, 10], and tune down immune surveillance mechanisms of the host [11]. In addition, they tune down immune-stimulating mechanisms of *Mtb*, if *Mtb* are able to acquire dormancy from the host [1, 4, 6].

Here, we detect *Mtb* DNA in human peripheral blood, selectively accumulated in a portion of CD34^+^CD90^+^CD38^-^ pHSCs [12, 13], as well as previously reported CD271^+^CD45^-^ mesenchymal stem cells (MSCs) [14, 15], from individuals with LTBI determined by IFN-γ release assay (IGRA) [16]. In mice that harbor *Mtb*, but do not develop TB, we, again, identified a portion of LT-pHSCs in bone marrow [17, 18] selectively infected with *Mtb*. *Mtb* in human and mouse pHSCs express stress or dormancy genes and do not form colonies on agar [19, 20]. Intratracheal application of human and mouse pHSCs harboring *Mtb* into immune-deficient mice resuscitates *Mtb* replication accompanied by increased cellularity indicative of an inflammatory infiltrate, typically observed when these mice are infected with replicating *Mtb*.

LT-pHSCs are thought to reside in hypoxic niches of bone marrow in close contact with other hematopoietic and non-hematopoietic cells, e.g. mesenchymal stem cells (MSCs) [7, 8, 21], that provide the supportive microenvironmental niche for HSCs [22]. *Das et al*. have reported that human bone marrow CD271^+^CD45^−^ MSCs *in vitro* as well as an equivalent population of bone marrow MSCs in the mouse may provide a long-term protective intracellular niche in the host in which *Mtb* can reside [23, 24]. The quiescence of the hypoxic niche of mesenchymal and hematopoietic cells could provide stress and induce dormancy genes of *Mtb* [25–27].

Our findings suggest that *Mtb*-infected bone marrow-derived hematopoietic stem cells of mice and humans, together with *Mtb*-infected MSCs serve as a hitherto unappreciated cellular niche during LTBI, capable of resuscitating active TB disease. *Mtb*-infected, blood-borne LT-pHSCs can be used to diagnose LTBI, and to monitor drug treatments. Their existence challenges current hypotheses of TB pathogenesis and epidemiology.

## Results

### Human peripheral blood SP^+^ and Lin^–^CD34^+^CD90^+^CD38^lo^ pHSCs of IGRA^+^ donors carry *Mtb* DNA

**Fig 1:**
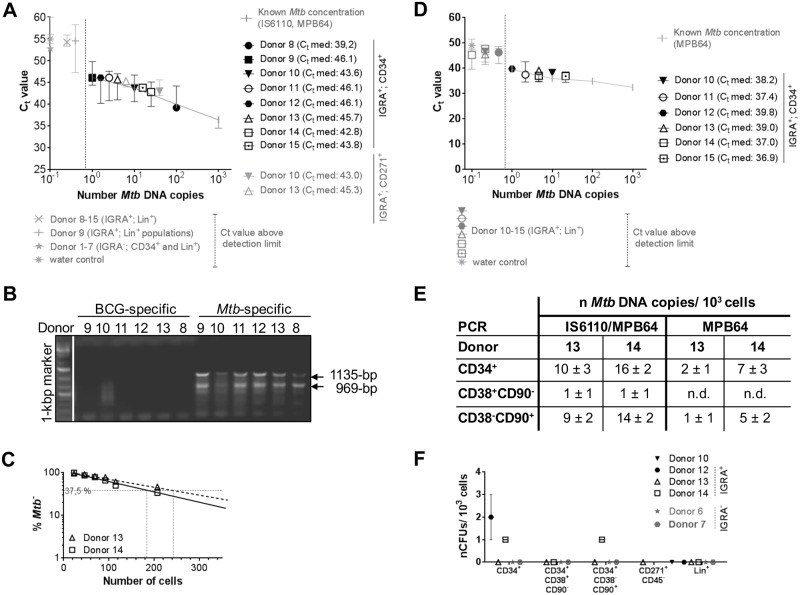
Human peripheral Lin^–^CD34^+^, Lin^–^CD34^+^CD38^low^CD90^+^, SP^+^ pHSCs as well as CD271^+^CD45^-^ MSCs of IGRA^+^ donors harbour *Mtb* DNA. Lin^+^, Lin^–^CD34^+^, Lin^–^CD34^+^CD38^low^CD90^+^, Lin^–^CD34^+^CD38^+^CD90^–^, Lin^–^SP^+^ and Lin^+^ SP^−^cells (Donors 1, 6, 8, 9) were purified from blood of IGRA^+^ (n = 8) and IGRA^−^donors (n = 7; S1A Fig). CD271^+^CD45^-^ MSCs (Donors 10 and 13) were purified from blood of IGRA^+^ donors (n = 2; S1B Fig). CD1c^+^, CD14^+^, CD16^+^, CD4^+^/8^+^, CD15^+^, CD19^+^, and CD56^+^ cells were prepared from blood of IGRA^+^ donors (n = 3). Genomic DNA prepared from 10^3^ hematopoietic progenitors and MSCs as well as 10^5^ Lin^+^ cells from IGRA^+^ and IGRA^-^ donors were tested for the presence of *Mtb* DNA by PCR. **(A, E)** Quantification of *Mtb*-specific DNA by real-time TaqMan PCR using probes that target *MPB64* and *IS6110* together (S4 Fig). **(B)** Genomic DNA of 10^3^ hematopoietic progenitors from IGRA^+^ and IGRA^-^ donors were tested by PCR for a DNA fragment present in *Mtb*, but not in BCG. **(C)** Quantification of *Mtb*-specific DNA by limiting dilutions using a single-target PCR for *IS6110* (Donor 13, 14; S2A Fig). **(D, E)** Quantification of *Mtb*-specific DNA by real-time SYBR green PCR using primers that target *MPB64* alone (S4 Fig). Real-time PCRs were performed in 2 independent runs in technical triplicates and normalized to human GAPDH. Known *Mtb* concentrations were used as reference. **(F)** CFU *Mtb* growth on Middlebrook 7H11 agar plates (n = 2–3). Data are shown as median + interquartile.

We searched for *Mtb* DNA in human pHSCs of donors with LTBI. To this end we purified Lin^–^CD34^+^ progenitors, and within them Lin^–^CD34^+^CD90^+^CD38^-^ pHSCs (S1A Fig), as well as, CD1c^+^ dendritic cells, CD14^+^CD16^low^ and CD14^low^CD16^+^ monocytes, CD15^+^ granulocytes, CD4^+^ or CD8^+^ T cells, CD19^+^ cells identifying B as well as dendritic cells and CD56^+^ NK cells by FACS from blood of IGRA^+^ and IGRA^−^donors (Table 1). We also isolated pHSCs by their drug efflux properties as Hoechst low/ negative side population (SP) phenotype cells (S1A Fig), since pHSCs are highly enriched in SP cells [28, 29]. DNA of all these potentially *Mtb*-infected cells was used to PCR-amplify DNA fragments of *Mtb*-encoded sequences to search for *Mtb* infection and Bacille Calmette-Guérin (BCG)-encoded sequences to score for possible remnants of a BCG-vaccination (Fig 1A–1E) [30, 31].

**Table 1:** Patient characteristics.

#	Age	Sex	IGRA	Comorbidities	Medication
**1**	22	m	neg	Psoriasis	-
**2**	53	f	neg	-	-
**3**	38	m	neg	-	-
**4**	33	m	neg	allerg RC	-
**5**	45	f	neg	-	-
**6**	38	f	neg	-	-
**7**	41	f	neg	-	-
**8**	29	m	0.87	Psoriasis	-
**9**	43	m	1.8	Psoriasis	-
**10**	27	f	10	Psoriasis	-
**11**	57	m	3.64	-	-
**12**	55	m	4.2	chronic Urticaria	Antihistamines
**13**	37	f	0.65	Eczema	-
**14**	46	f	2.71	-	-
**15**	48	m	9	-	-

Tablenotes: #: patient number; Sex—m: male; Sex—f: female; neg: negative; allerg RC: allergic rhinoconjunctivitis

Eight of eight IGRA^+^ donors scored positive in blood cells for *Mtb* (Fig 1A–1E), and none of them positive for BCG (Donors 8–13; Fig 1B), selectively in ~2×10^3^ SP^+^ and Lin^–^CD34^+^ pHSCs. Quantitation of *Mtb*-specific DNA was done by real-time TaqMan PCR targeting two *Mtb*-specific genes, the single copy *MPB64* and the multiple copy *IS6110* sequence (Fig 1A and 1E), as well as in limiting dilution PCRs targeting *IS6110* alone (Fig 1C; S2A Fig). In PCR tests detecting multiple *IS6110* elements in a single *Mtb* genome [32–35], in SP^+^ and Lin^–^CD34^+^ pHSCs from IGRA^+^ donors we detected between seven and twenty copies of *Mtb*-specific DNA within lysates of 10^3^ cells (Fig 1A, 1C and 1E; S4 Fig). Using primers that target the single copy *MPB64* alone, we detected between one and seven copies of *Mtb* DNA within lysates of 10^3^ cells (Fig 1D; S4 Fig). In the genome of individual IGRA^+^ *Mtb*^+^ donors two to 10-fold higher *IS6110* copy numbers were detected than the one *MPB64* copy.

We also analyzed CD271^+^CD45^-^ MSCs (S1B Fig) from selected donors (Donors 10 and 13) for presence of *Mtb*. Thereby, we detected between one and ten copies of *Mtb*-specific DNA within lysates of 10^3^ MSCs by real-time TaqMan PCR targeting *MPB64* and *IS6110* from blood of human donors with LTBI (Fig 1A), in confirmation of previous results by *Das et al*. [23, 24]

Moreover, none of the IGRA^−^donors harbored detectable *Mtb* DNA in any of the Lin^−^and Lin^+^ blood cells tested by both PCRs (Fig 1A and 1D). From two IGRA^+^ donors, CD34^+^ progenitors were further FACS-purified as Lin^–^CD34^+^CD90^+^CD38^lo^ pHSCs (S1A Fig) [13]. 10^3^ of these harbored between 9 and 14 *Mtb* DNA copies in the qPCR targeting *MPB64* and *IS6110*, and between one and five in the *MPB64* qPCR, while 10^3^ of the more differentiated Lin^–^CD34^+^CD38^+^CD90^–^ cells harbored none (Fig 1E). Also, 10^3^ of the pool of Lin^+^ cells of IGRA^+^ donors, as well as FACS-purified dendritic cells, monocytes, granulocytes, T cells, B cells and NK cells, scored negative in all of these qPCR assays (Fig 1A and 1D).

We conclude that within human peripheral Lin^-^SP^+^ and CD34^+^ cells in blood of IGRA^+^ donors, the Lin^–^CD34^+^CD90^+^CD38^-^ pHSCs, as well as CD271^+^CD45^-^ MSCs, selectively carried *Mtb* DNA while their peripheral Lin^+^ cells were consistently *Mtb*^–^.

Replication-competent *Mtb* can form colonies on agar. Thus, we tested the different cell populations from three IGRA^+^ donors for growth measured by enumerating colony-forming units. Thereby, only one CFU was formed from lysates of 10^3^ Lin^–^CD34^+^ and Lin^–^CD34^+^CD90^+^CD38^-^ pHSCs isolated from two of the donors, which, in the *MPB64* PCR assays, contained between one and five *Mtb* DNA copies (Fig 1A–1F). Thus, potentially only one of five *Mtb* DNA copies detected in Lin^–^CD34^+^ and Lin^–^CD34^+^CD90^+^CD38^-^ cells was replication-competent, the majority were non-replicative in quiescent hematopoietic cells. Furthermore, from lysates of 10^3^ CD271^+^CD45^-^ MSCs no CFUs were detected (Fig 1F). Therefore, in the donors tested the majority of *Mtb* DNA copies detected in CD271^+^CD45^-^ MSCs cells were similarly not replication-competent.

### A portion of CD150^+^ LT-pHSCs, but neither ST-pHSCs nor MPPs in bone marrow of *Mtb*-infected mice harbour *Mtb* DNA

**Fig 2:**
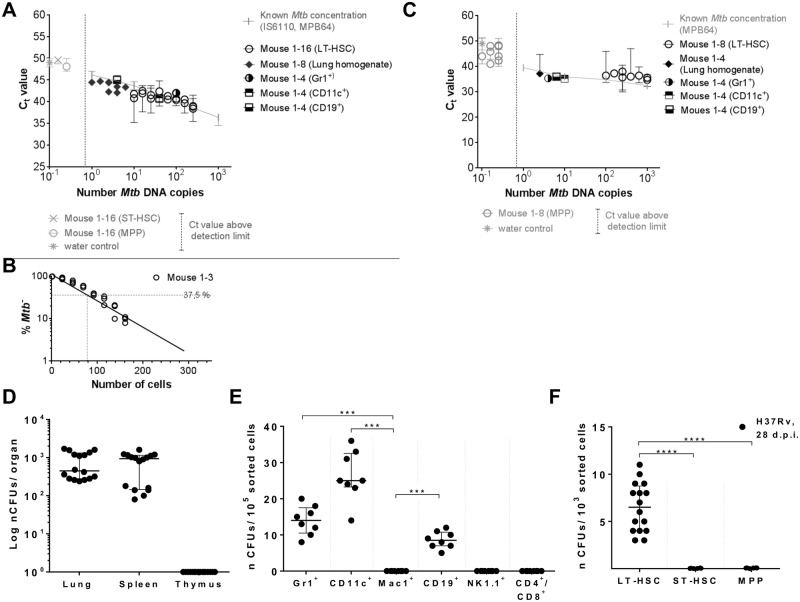
Detection of *Mtb* infection in different organs and hematopoietic cells of mice day 28 p.i. by *Mtb* DNA PCR and *Mtb* CFU. C57BL/6 mice were infected with 10^5^ CFUs *Mtb* (H37Rv). **(A)** Quantification of *Mtb*-specific DNA by real-time TaqMan PCR using probes targeting *MPB64* and *IS6110* (S4 Fig) on genomic DNA of 10^5^ lung cells (n = 8), 10^5^ Gr1^+^, CD11c^+^, CD19^+^, Mac1^+^, NK1.1^+^, CD4^+^/8^+^ cells (n = 4; S1C Fig), and 10^3^ LT-pHSCs, ST-pHSCs and MPPs (n = 16; S1B Fig). **(B)** Quantification of *Mtb*-specific DNA by limiting dilutions using a single-target PCR for *IS6110* (n = 3; S2B Fig). **(C)** Real-time SYBR green PCR using primers targeting *MPB64* (n = 4–8; S4 Fig). Real-time PCRs were performed in 2 independent runs in technical triplicates and normalized to murine GAPDH. Known *Mtb* concentrations were used as reference. **(D)** CFU enumeration on Middlebrook 7H11 agar in cells of lung, spleen and thymus (n = 16). **(E)** CFU enumeration on Middlebrook 7H11 agar for Lin^+^ cell populations (n = 8). **(F)** CFU enumeration on Middlebrook 7H11 agar for hematopoietic progenitors (n = 16). Shown are data of 4 independent experiments. Data are shown as median + interquartile. *P ˂ 0.05, **P ˂ 0.005, ***P ˂ 0.0005, ****P ˂ 0.00005 by Mann-Whitney test.

Next, we infected mice with *Mtb* to see whether bone marrow-derived LT-pHSCs could become carriers of the bacterium too. We used a mouse model of intradermal ear infection, where low numbers of *Mtb* persist systemically without developing active TB typically seen after aerosol infection of mice with *Mtb* [5]. A variety of organs, such as the lung, and the spleen, but not the thymus, and hematopoietic cells in them were found to be infected 28 days post-infection (p.i.). DNA purified from 10^5^ lung cells harboured between one and ten copies of *Mtb* DNA (Fig 2A and 2C).

10^3^ FACS-purified Lin^–^Sca1^+^c-Kit^+^CD150^+^CD48^–^ LT-pHSCs (S1C Fig) [36] were found to harbor between 40 and 100 copies of *Mtb* DNA, as detected by qPCR targeting *MPB64* and *IS6110* and in limiting dilution analyses targeting *IS6110* alone (Fig 2A–2B; S2B Fig). In *MPB64* qPCRs LT-pHSCs were found to harbor between five and 90 copies of *Mtb* DNA (Fig 2C). By contrast, in 10^3^ Lin^–^Sca1^+^c-Kit^+^CD150^+^CD48^+^ short-term repopulating pluripotent hematopoietic stem cells (ST-pHSCs) and Lin^–^Sca1^+^c-Kit^+^CD150^-^CD48^+^ multipotent progenitors (MPPs) (S1C Fig) [36], no *Mtb* DNA could be detected using any of these PCR analyses (Fig 2A and 2C).

Furthermore, no *Mtb* DNA was found in 10^5^ FACS-enriched Mac1^+^ macrophages, NK1.1^+^ NK cells, and CD4^+^ as well as CD8^+^ T cells (Fig 2A; S1D Fig), while between eight and 60 copies of *Mtb* DNA were found in qPCR analyses of lysates of 10^5^ FACS-enriched CD11c^+^ dendritic cells, Gr1^+^ granulocytes and CD19^+^ cells identifying B as well as dendritic cells, representing 10 to 100-fold lower numbers of *Mtb* DNA copies than in LT-pHSCs (Fig 2A and 2C). We have not attempted to purify MSCs from bone marrow of infected mice.

We conclude that intradermal infection of mice resulted in *Mtb*-infected LT-pHSCs in bone marrow 28 days p.i., and that during this infection, similarly to our observations in human LTBI, only LT-pHSCs harbored *Mtb* DNA among specific pHSC populations. Nevertheless, in this mouse model, the lung and more mature Lin^+^ cells in spleen and bone marrow of mice are infected with *Mtb* detected by qPCR and CFU assays, which is in contrast with our observations in human LTBI. Accordingly, we do not suggest that this mouse infection model, at day 28 p.i., is comparable to the pathophysiological status of latent infections in LTBI. Nevertheless, the selective *Mtb* infection of LT-pHSCs over ST-pHSCs and MPPs was recapitulated in our mouse model.

### Numbers of replication-competent, colony-forming *Mtb* in *Mtb*-infected mouse cells

We tested the different *Mtb*- DNA^+^ cell populations for replication-competent, active *Mtb* measured by enumerating CFUs (Fig 2D–2F). The vast majority, if not all, of the *Mtb* bacteria detected by PCR in 10^5^ lung cells (Fig 2A, 2C and 2D), as well as in 10^5^ FACS-enriched CD11c^+^ dendritic cells, Gr1^+^ granulocytes and CD19^+^ dendritic or B-lineage cells in bone marrow (Fig 2A, 2C and 2E) produced CFUs. Hence, most *Mtb* bacteria inside these cells were replication-competent. These results also document, that the two assays, for *Mtb* DNA and for CFUs of active *Mtb*, detect comparable numbers of bacteria.

By contrast, only 10 CFUs were formed from lysates of 10^3^ LT-pHSCs isolated from *Mtb*-infected mice, which, in *MPB64* PCR assays, contained between five and 90 *Mtb* DNA copies (Fig 2C and 2F). Thus, up to 80% of *Mtb* DNA in LT-pHSCs were not replication-competent as assayed by CFUs.

Our results also suggest that not all, but only a part, of the LT-pHSC pool is infected by *Mtb*, almost all of which were in a quiescent stage. However, these analyses do not reveal the number of infected cells in the pHSC pool.

**Fig 3:**
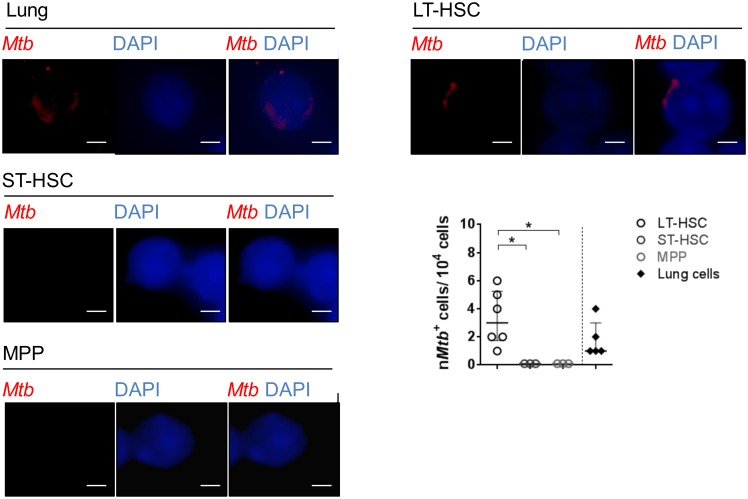
Detection of *Mtb* in cells of the lung and hematopoietic cells of *Mtb*-infected mice by histology. Rhodamin-auramin stainings of representative LT-pHSCs (n = 6), ST-pHSCs (n = 3), MPPs (n = 3) as well as cells of the lung (n = 5) at day 28 p.i. For each sample (cell sort) 10,000 cells were screened per slide for *Mtb* positive cells. At least 3 images were taken from each slide of each sample. Rhodamin-auramin stainings were screened on high power (100×) and verified under oil immersion using a fluorescent microscope. Analyses were carried out using ProGres Capture Pro 2.8.8. (*Mtb*, red; nuclei, blue). Shown are representative data (cropping of images) for staining of LT-pHSCs, ST-pHSCs, MPPs and cells of the lung. scale bar: 10 μm. *P ˂ 0.05 by Mann-Whitney test.

**Fig 4:**
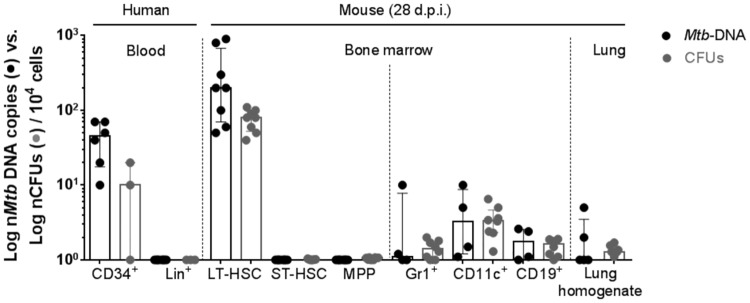
Numbers of *Mtb* DNA copies (*MPB64* qPCR) and CFUs in 10^4^ cells of different cell populations. Data are shown as median + interquartile (n = 4–8).

Next we directly visualized LT-pHSCs carrying *Mtb* by rhodamine-auramin staining [37]. In LT-pHSCs, *Mtb* was readily detectable, whereas ST-pHSCs and MPPs did not show positive staining (Fig 3). Approximately one to six of ~ 10^3^ analyzed LT-pHSCs stained positive for *Mtb* (Fig 3). As a control *Mtb*-infected lung cells were analyzed. In these cells the number of *Mtb* positive cells as revealed by rhodamine-auramin staining, qPCR and CFU were showing a good correlation (Figs 3 and 4).

However, this staining method was not as sensitive as qPCR in determining the actual number of LT-pHSCs harbouring *Mtb* [38].

### *Mtb* residing within human CD34^+^ as well as mouse CD150^+^ pHSCs express genes of the dormancy regulon

Since 10^3^ mouse LT-pHSCs were found to contain between five and 90 copies of *Mtb* genomes, but generated only 10 CFUs (Figs 2C, 2F and 4), we concluded that the vast majority, i.e. around 80%, of *Mtb* bacterial genomes persist in a non-replicating form. Dormancy or stress of *Mtb* is induced under hypoxic conditions and is reflected in a change of gene expression [25–27]. It is controlled by the dormancy regulon and involves transcription of approximately 50 so-called dormancy genes, among them *DosR*, *c-lat* and *hspX* [19, 25–27]. *SigA* is expressed in non-replicating as well as in replicating, CFU-forming *Mtb* and thus, can be used as a housekeeping gene [20].

We hypothesized that the hypoxic niche of LT-pHSCs, by being stressed, could induce dormancy of *Mtb* [7, 8]. To test for this hypothesis, we performed quantitative RNA expression analyses for *Mtb* dormancy genes in mouse LT-pHSCs as well as in *Mtb*-infected lung cells 28 days p.i., in which the vast majority of *Mtb* organisms form CFUs (Fig 4).

**Fig 5:**
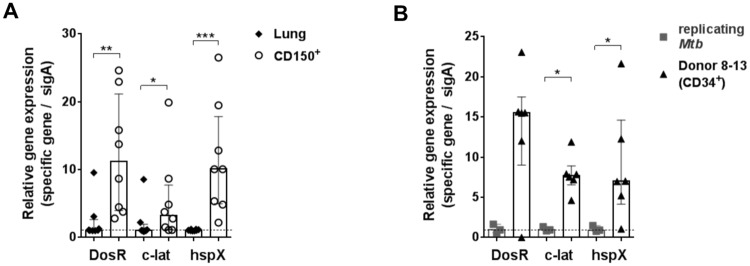
Murine and human pHSCs are infected with *Mtb* expressing dormancy genes. **(A)** Expression analyses on RNA isolated from *Mtb*-infected mouse lung cells and purified LT-pHSCs (n = 8). **(B)** Expression analyses on RNA isolated from Lin^–^CD34^+^ pHSCs from IGRA^+^ donors (n = 6) and *Mtb*-infected human monocytic leukemia cell line 96 h p.i. (n = 3). Expression analyses were done by real-time TaqMan PCR for *SigA*, *DosR*, *c-lat* and *hspX*. *SigA* was used as reference for *Mtb*. Real-time TaqMan PCRs were performed in 3 independent runs in technical triplicates. Data are shown as median + interquartile. *P ˂ 0.05, **P ˂ 0.005, ***P ˂ 0.005 by Mann-Whitney test.

*SigA* was detected in both cell types. By contrast, *Mtb* DNA^+^ LT-pHSCs did express *DosR*, *c-lat* and *hspX* RNA, while *Mtb* from infected lung cells did not express dormancy genes (Fig 5A). We conclude that *Mtb* resides within mouse CD150^+^ LT-pHSCs in a non-replicating state, expressing dormancy genes.

In human *Mtb*-infectedSP^+^ and Lin^–^CD34^+^ pHSCs we similarly detected both *SigA* expression and the expression of the dormancy regulator genes, *DosR*, *c-lat* and *hspX*, while replicating *Mtb* isolated from an infected human monocytic leukemia cell line did not express these genes (Fig 5B). Collectively, our results show that pHSCs act as an intracellular niche for stressed or dormant non-replicating *Mtb* in mice and humans.

### Intratracheal transfer of *Mtb*-infected human and murine pHSCs leads to resuscitation and expansion of active TB in transplanted hosts

**Fig 6:**
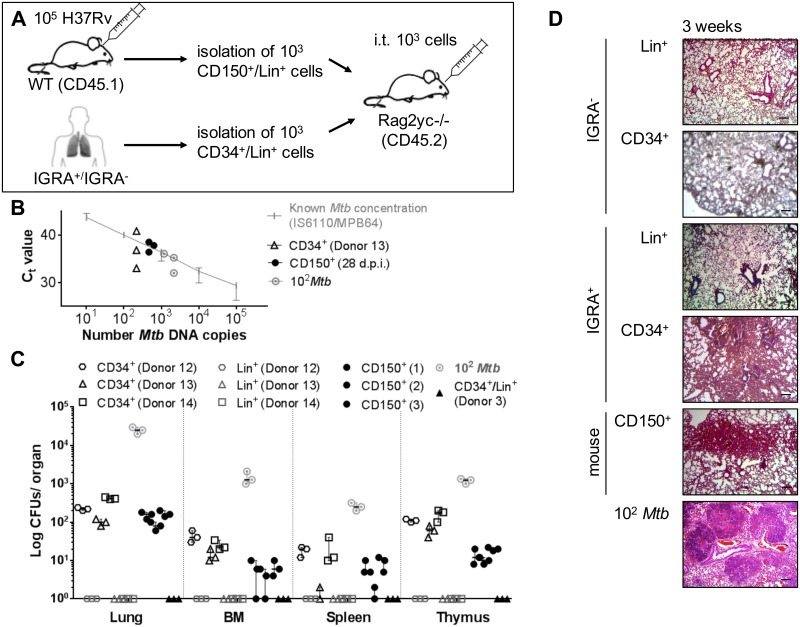
Intratracheal transfer of *Mtb* infected human and murine pHSCs leads to *Mtb* growth and increased cellularity into the lungs in transplanted hosts. **(A)** Injection of Lin^-^CD34^+^ and Lin^+^ cells from blood of IGRA^+^ human donors (Donor 12–14) and mouse LT-pHSCs from bone marrow 28 days p.i. into the trachea of *Rag2*^–/–^*Il2rg*^/–^mice (3 mice/population). Transfer of 10^2^ CFUs *Mtb* was used as positive (n = 3), uninfected pHSCs and Lin^+^ cells of an IGRA^−^donor (Donor 3; n = 1) as negative, control. Recipients were analyzed after 3 weeks. **(B)** Monitoring of *Mtb* infection by TaqMan PCR using probes that target *MPB64* and *IS6110* together on genomic DNA of 10^5^ lung cells 3 weeks upon transfer. PCRs were performed in technical triplicates and normalized to murine GAPDH. **(C)** CFU *Mtb* growth on Middlebrook 7H11 agar in cells of lung, spleen, thymus and non-separated, 10^5^ bone marrow cells 3 weeks upon transfer (n = 3/population). Shown are data from 3 independent experiments. **(D)** Histopathology of representative lung sections 3 weeks upon transfer. Lungs were stained with hematoxylin/eosin, screened with 5×objectives and verified using a light microscope. Shown are representative data from 3 independent experiments. Data are shown as median + interquartile. Scale bar: 100 μm.

Finally, we tested the capacity of *Mtb*-infected pHSCs of human LTBI and of mice 28 days p.i. to resuscitate active infection upon intratracheal application into the trachea of *Rag2*^–/–^*Il2rg*^–/–^mice [39]. One thousand Lin^–^CD34^+^ cells from blood of latently infected, IGRA^+^, *Mtb* DNA^+^ human donors, containing between 1 and 7 copies of *Mtb*-DNA, and of IGRA^−^donors (Fig 1A and 1D; S4 Fig), Lin^–^CD150^+^CD48^–^ LT-pHSCs from bone marrow of infected mice (Fig 2A and 2C), containing between 5 and 90 (Fig 2C) copies of *Mtb*-DNA as well as 100 pure, replication-competent *Mtb* as control, were administered into the trachea of *Rag2*^–/–^*Il2rg*^–/–^mice and organs were analyzed 3 weeks later (Fig 6A). *Mtb* DNA as well as active, replicating *Mtb* CFUs were detected in the lungs (Fig 6B and 6C). Between 100 and 400 CFUs were detected in total lung cells of mice receiving human LT-pHSCs, while in lungs of mice receiving mouse LT-pHSCs contained between 50 and 200 CFUs. Hence, *Mtb* contained in pHSCs had expanded between 10 and 100-fold as active, replicating bacteria. Active, replicating *Mtb* was also detected in spleen, thymus, and bone marrow in mice recieving pHSCs from IGRA^+^ donors and *Mtb*-infected mice, but not in mice recieving Lin^+^ cells from IGRA^+^ donors or cells from IGRA^−^donors (Fig 6B–6C).

We conclude from these results, that *Mtb* replicated in the lungs of mice, into which *Mtb*-infected human or mouse pHSCs had been transferred. These expanded *Mtb* were replication—competent and had spread systemically. However, while in total bone marrow cells of the recipient mice *Mtb* DNA and replicating *Mtb* CFUs were detectable (Fig 6C), pHSCs of the donors (human or mice) could not be detected by FACS in the bone marrow of the recipient. This suggests, that bone marrow infection was caused by a dissemination of replication-competent *Mtb* from the lung resulting from a primary infection, rather than by *Mtb*-infected donor pHSCs homing from lung to bone marrow.

In histological sections of lungs of the *Rag2*^–/–^*Il2rg*^–/–^mice transplanted with either human pHSCs from LTBI or mouse pHSCs from *Mtb*-infected mice, we observed increased cellularity in the lungs indicative of an inflammatory infiltrate in response to an active infection 3 weeks after pHSC transfer (Fig 6D). Transfer of Lin^+^ cells from IGRA^+^ donors and cells from IGRA^−^donors did not induce these histological changes in the lung of recipients.

The observed increase in cellularity in the lung could result from the expansion of replication-competent *Mtb* or from both replication-competent and stressed dormant bacteria. In the latter case, stressed dormant *Mtb* could be resuscitated to active replicating bacteria. In any case, we conclude that *Mtb*-infected human and mouse pHSCs can reproduce an active infection after introduction into recipient mice by intratracheal transfer.

## Discussion

**Fig 7:**
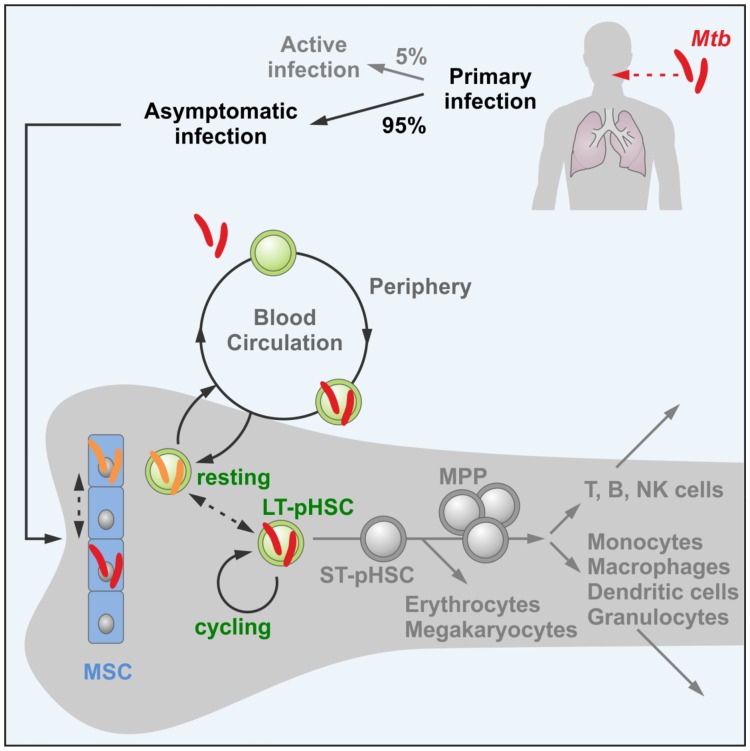
A model of a long-term persisting niche for non-replicating *Mtb* bearing the risk of resuscitation of active *Mtb*. Model of LTBI where non-replicating *Mtb* reside, and perhaps move, between long-lived, resting hematopoietic and non-hematopoietic cells in hypoxic niches in bone marrow and in which actively replicating *Mtb* can be resuscitated leading to TB.

Our results suggest that, in individuals with LTBI, dormant *Mtb* bacteria reside, and perhaps transit, between long-lived, resting hematopoietic and non-hematopoietic cells in hypoxic niches (Fig 7) [6–8, 40]. We have interpreted the expression of *DosR*, *hspX* and *c-Lat* genes as a sign of either stress or dormancy of *Mtb*, both induced in hypoxic areas of bone marrow that are thought to promote energy saving, and thus, could favor long-term rest of both LT-pHSCs and *Mtb*. If so, do the energy-saving gene expression programs of both *Mtb* and host pHSCs interact with each other [4, 19]?

Future whole transcriptome next-generation sequencing of *Mtb*- as well as of LT-pHSC-encoded genes expressed in single LT-pHSCs will not only allow monitoring of *Mtb*- but also of LT-pHSC-controlled gene expression programs and their potential for interactions in hypoxic stem cell niches of bone marrow. It will also provide more precise information on the number of LT-pHSCs infected by either replicative or dormant *Mtb*. If *Mtb* and LT-pHSCs adapt to each other by dormancy, it is conceivable that other facultative intracellular bacteria could find the same long-term quiescent niche for long-term persistence in a dormant state.

The possibility of a transfer of *Mtb* from infected bone marrow donors has been made likely in several case reports that have described incidence of *Mtb* infections between 120 days and 20 months post allogenic bone marrow transfer. However, these reports only refer to the induction of an *Mtb* infection as a consequence of the administration of immunosuppressive drugs to the recipients [41–45]. While patients that receive an allogeneic pHSC transfer, or that are scheduled to be treated with anti-inflammatory agents such as anti-TNFα antibodies, are usually tested for their IGRA-status prior immunosuppressive treatment, pHSCs are not screened for possible infections. Our results suggest that bone marrow donors should be screened for *Mtb* infection, so that they can be cured of the infection prior to bone marrow transplantation.

Within the limited numbers of human donors with LTBI, that were available to us, all of them carried *Mtb* exclusively in pHSCs. Two of them were also analysed for the presence of *Mtb* in MSCs, and both were positive. However, a much larger number of LTBI donors should be screened to evaluate, whether a small percentage of LTBI donors could be free of *Mtb* in their pHSCs. Such a larger analysis could also test the possibility, that a low percentage of LTBI donors could carry *Mtb* in the progeny of pHSCs, e.g. in MPPs, CMPs or CLPs.

A host with LTBI has immunological memory for *Mtb* [2]. Therefore, resuscitation of TB will only be successful if the immune system of the LTBI host fails to eliminate cells, in which actively replicating *Mtb* have been resuscitated from a stressed or dormant state. This can be readily observed in patients with inborn or acquired immunodeficiencies, e.g. in HIV-infected patients or patients treated with TNFα-inhibitors. The precise stimuli leading to reactivation require further investigation. However, the consequences of pHSCs spreading active *Mtb* throughout the body, either directly or after differentiation, can be detrimental. Our data thereby provide an additional explanation for the possible occurrence of reactivated TB in other bodily organs after primary infection and encapsulation in granulomas.

## Materials and Methods

### Human samples

Latently *Mtb* infected subjects included in the study were from a Western country, had not been treated previously for tuberculosis (TB), had normal chest radiography and were not suffering from active TB. Hence, LTBI individuals were routinely identified by positive IGRA (Quantiferon-TB Gold^®^ test, Cellestis, Qiagen) and exclusion of active TB. IGRA testing was performed either because of a scheduled treatment with TNF-α inhibitors or because of occupational contact with patients suffering from active pulmonary TB.

Collection of blood samples was approved by the Ethics Committee of the Medical University of Vienna (EK 071/2005) and conducted according to the Declaration of Helsinki. Informed written consent was obtained from all patients.

### Mice

C57BL/6 wild-type mice were purchased from Charles River Laboratories. CD45.1 C57BL/6 and *Rag2*^–/–^*Il2rg*^–/–^mice were bred in our facilities. Infected mice were maintained at biosafety level 3. All animal experiments were approved by the local ethics committee of the German authorities (State Office of Health and Social Affairs Berlin; *Landesamtes für Gesundheit und Soziales Berlin*, # G0009-14).

### Infection with *Mtb*

*Mtb* strain H37Rv was cultured in Middlebrook 7H9 broth (BD) supplemented with 0.05% (v/v) Tween 80 and Middlebrook AODC Enrichment (BD) to mid-log phase (OD _600 nm_ 0.6–0.8). Bacteria were harvested, resuspended in PBS (GIBCO), and frozen at –80°C until use. For dermal infections, 8- to 10-week-old female C57BL/6 wild-type mice were anesthestized by i.p. administration of ketamine (50 mg/kg) and Rompun (5 mg/kg; Bayer), and 10^5^ *Mtb* in 50 μl PBS were administered into the ear dermis. Mice were monitored daily regarding their health, body condition and well-being. Specifically, we monitored mice for loss of body weight, abdominal respiration and lesions of ear dermis. Once a week mice were weighed. At the end of experiment mice were sacrificed by cervical dislocation. For the infection of human monocytic leukemia cells *in vitro*, THP-1 cells (ATCC^®^TIB-202^™^, ATCC cell lines, UK) were used, that were authenticated by STR profiling and tested for mycoplasma contamination. We have not used any cell line from the list of commonly misidentified cell lines (ICLAC). THP-1 cells were seeded in T_75_ flasks (TPP) in complete RPMI-1640 (cRPMI, RPMI-1640 medium supplemented with 1% L-glutamine, 1% Hepes, 0.1% 2-ME and fetal bovine serum to a final concentration of 10%; GIBCO, Life Technologies). For proper viability of cells, a concentration of 1 × 10^6^ cells/ml was not exceeded. Cells were incubated at 37°C and 5% CO_2_. Differentiation to macrophages was triggered by overnight incubation with PMA (50 ng/ml), followed by two washes in RPMI-1640 and addition of cRPMI-1640 over 48 h post-differentiation. For infection, 10^7^ differentiated macrophages were seeded into T_150_ flasks in 25 ml cRPMI and 1 ml of medium containing 10^5^ *Mtb* was added. Non-internalized bacteria were washed away 4 h p.i. using PBS and cells were placed back in cRPMI. Cells were harvested for RNA isolation 48 and 96 hours p.i.

### Antibodies

For the purification of 2–40 × 10^3^ circulating human hematopoietic precursor cells as well as mesenchymal stem cells from 90 ml of peripheral blood, PBMCs were obtained by Ficoll-Paque density gradient centrifugation (Ficoll-Paque Plus; GE Healthcare Bio-Sciences AB, Uppsala, Sweden) and the following antibodies were used: CD1c (clone AD5-8E7, Miltenyi Biotec), CD3 (UCHT1, Beckman Coulter), CD11c (Bu15, Beckman Coulter), CD14 (RMO52, Beckman Coulter), CD15 (HI98, BioLegend), CD16 (3G8, Beckman Coulter), CD20 (2H7, BioLegend), CD41 (SZ22, Beckman Coulter), CD56 (C218, Beckman Coulter), CD203c (NP4D6, BioLegend), CD235a (KC16, Beckman Coulter), BDCA2 (AC144, Miltenyi Biotec), CD34 (8G12, BD Biosciences), CD38 (HIT2, Biolegend), CD90 (5E10, Biolegend), CD45 (H130, BD Bioscience) and CD271 (ME20.4-1.H4, BD Bioscience). Secondary staining was done with goat anti-mouse IgG Alexa Fluor 488 (MolecularProbes). Immunomagnetic depletion was performed using anti-mouse IgG (magnetic cell sorting; Miltenyi Biotec). Hematopoietic progenitor cells were sorted to a purity >98% on a FACS Aria (BD Biosciences).

For purification of 10^3^ cells of mouse bone marrow hematopoietic progenitor cells and 10^5^ Lin^+^ cells, the following antibodies were used: Mac1 (M1/70), Gr1 (RB6-8C5), Ter119 (TER-119), CD19 (1D3), B220 (RA3-6B2), CD5 (53–7.3), CD3ε (145-2C11), CD11c (N418), CD4 (GK1.5), CD8 (53–6.7), NK1.1 (PK136), c-Kit (2B8), Sca1 (D7), CD150 (TC15-12F12.2) and CD48 (HM48-1). Antibodies were obtained from eBioscience. Cells were sort purified to a purity of >98% on an LSRII flow cytometer (Aria II, BD Biosciences).

### Hoechst staining

Human PBMC were resuspended in SP buffer (HBSS, 2% FCS, 2mM HEPES buffer; GIBCO, Life Technologies), prewarmed to 37°C and incubated with Hoechst 33342 (Molecular Probes, Life Technologies) at 5 μg/ml for 2 h at 37°C. All subsequent steps were carried out on ice. Cells were stained with antibodies against lineage markers as described above. 7-AAD (5 μg/ml, Calbiochem) was added for live—dead cell discrimination. As negative control, PBMCs were preincubated with verapamil (100 μM, Sigma Aldrich). Cells showing a dim staining in the Hoechst blue (450/50 nm band pass filter) and Hoechst red (660/20 nm) channels were sorted to a purity >98% on a FACS ARIA (BD Biosciences).

### *Mtb* DNA detection

We used the mycobacterial DNA extraction procedure first described by *van Soolingen et al*. 50 ng (pHSCs)—1 μg (Lin^+^ and lung cells) of DNA was analyzed by real-time TaqMan^®^ PCR using gene-specific probes targeting two *Mtb* sequences, namely *MPB64* and *IS6110* together (Path-M.tuberculosis_*MPB64*/*IS6110*, Integrated Science). Each sample was assayed in technical triplicates. TaqMan probes for GAPDH were used as endogenous controls for eukaryotic cells (Human: Hs99999905_m1, Mouse: Mm99999915_g1, Invitrogen). H37Rv DNA was used to construct a standard curve for the probes.

In addition, DNA was analyzed by quantitative PCR using the primers 5′-CAGGCATCGTCGTCAGCAGC-3′ and 5′-GTGATTGGCTTGCGATAGGC-3′ targeting *MPB64* alone (543-bp DNA fragment) [46], using the SYBR green system of detection. Primers for human 5′-CTCCCCACACACATGCACTTA-3′ and 5′- CCTAGTCCCAGGGCTTTGATT-3′ and mouse *GAPDH* 5′-CATGTTCCAATATGATTCCAC-3′ and 5′-CCTGGAAGATGGTGATG-3′ were used as endogenous controls for eukaryotic cells. H37Rv DNA was used to construct a standard curve for primers used. PCR products were detected as an increase in fluorescence with the ABI PRISM 7700 instrument and quantified using the SDS software, version 2.2.2.

In order to reduce amplification backgrounds with primers, we have performed quantitative PCR analyses using a “no template = water control” for every run. Exponential amplification in the “water control” with Ct values of 51–55 was taken as the detection limit for *Mtb* in PCRs targeting *IS6110* and *MPB64* together. In PCRs targeting *MPB64* alone, “water control” Ct values of 48–50 was taken as the detection limit. To ensure that this background did not result from a contamination by genomic *Mtb* DNA, the *MPB64* qPCR product was analyzed by gel electrophoresis. While the expected PCR product size was detectable in the *Mtb*^+^ samples, such a distinct PCR band were not found in the “water control” (S3 Fig). In PCRs targeting *IS6110* together with *MPB64* we considered samples as positive for *Mtb* with a Ct value of 48 (equivalent to 1 *Mtb* DNA copy) or lower (equivalent to several *Mtb* DNA copies). In PCR tests targeting *MPB64* alone, we considered samples as *Mtb* positive with a Ct of 39–40 (equivalent to 1 *Mtb* DNA copy) or lower (equivalent to several *Mtb* DNA copies).

For limiting dilution analyses on DNA the primers 5′-CGTGAGGGCATCGAGGTGGC-3′ and 5′-GCGTAGGCGTCGGTGACAAA-3′ were used to amplify a 245-bp DNA fragment encoded by the *IS6110* insertion sequence in the *Mtb* genome [30]. At the point where the PCR signal was lost in serial dilutions, limiting dilution analyses were performed.

*Mtb*-specific and BCG-specific DNA was also detected using primers previously described [31]. PCR reactions were performed in a thermal cycler at 95°C for 15 min, followed by 50 cycles at 95°C for 30 s, 45 s at different annealing temperatures and 45 s at 72°C (DNA Engine^®^ PTC2000, Biozym DiagnosticRad). For every reaction uninfected DNA and DNA from H37Rv *Mtb* were included. PCR products were analyzed by electrophoresis on 2% agarose gels.

### Colony-forming units

Mice were sacrificed at 28 days p.i., and organs (spleen, thymus, lung and bone marrow) were aseptically removed and homogenized in 1 ml PBS containing 0.05% Tween 80 (v/v). For pulmonary CFU determination, the lung was removed and incubated in 1 mg/ml collagenase type VIII (Sigma-Aldrich) and 30 μg/ml DNase I (Roche) at 37°C for 30 min. One half of each of the lung homogenate, the spleen, and the thymus were diluted in PBS containing 0.05% v/v Tween 80 and plated onto Middlebrook 7H11 agar plates supplemented with Middlebrook OADC Enrichment (Dibco). In addition, purified human and mouse pHSCs were plated. CFUs were enumerated after 4–6 weeks of incubation at 37°C and 5% CO_2_.

### RNA/qRT-PCR

Cells were homogenized in TRIzol (Invitrogen) and RNA was isolated via chloroform extraction (Life Technologies), treated with ethanol and dissolved in RNase-free water. RNA from *Mtb* infected THP-1 cells was isolated as previously described [47, 48]. One hundred ng of total RNA was reverse-transcribed by SuperScript III (Invitrogen) primed with oligodT. The cDNA for the specific target assays was then amplified by pre-amplification reaction using pooled gene-specific primers according to the manufacturer`s protocol (Invitrogen). The pre-amplification product was diluted [1:20) and finally analyzed by real-time TaqMan^®^ PCR using the following TaqMan probes: *DosR*, *c-lat*, *hspX* and *SigA* (Design Batch ID: w1406535517000, order number: 2106064SO, Invitrogen). *DosR*, *c-lat* and *hspX* RNA abundances were normalized to *SigA* as endogenous controls for *Mtb*. Each sample was assayed in triplicate. H37Rv DNA was used to construct a standard curve for all inspected genes. The PCR product was detected as an increase in fluorescence with the ABI PRISM 7700 instrument. RNA was quantified using the SDS software, version 2.2.2.

### Cytology

Sorted cells were fixed in PBS containing 4% w/v PFA for 24 h at 4°C. Thereafter, cells were immobilized by cytospin on a solid support (Shandon Centrifuge, Modell Cytospin 3). Slides were flooded with auramine-rhodamine for 15 min, fluorescent decolorizer for 2–3 min and potassium permanganate for 3–4 min. A 2-μg/ml working solution of DAPI was used for nuclear visualization.

Cover slips were mounted in ProLong Gold anti-fade reagent (Cat. No. P36934; Invitrogen) and sealed using adhesives. Slides were screened with 100× (for images) objectives under oil immersion using a fluorescence microscope (DMRB Fluorescence Microscope, Leica Microsystems). Analyses using the fluorescence microscope were done using ProGres Capture Pro 2.8.8. (Optical Systems, Jenoptick AG). For confocal microscopy image acquisition was performed using Zen 2010 Version 6.0 and images were analyzed by Zen 2012 Light Edition software (Carl Zeiss MicroImaging). For each sample 10,000 cells were analyzed per slide for the number of *Mtb* positive cells. For each slide at least three images of representative cells were taken.

### Histology

For histology, lung caudal lobes were preserved in PBS containing 4% w/v PFA for 24 h at 4°C, embedded in paraffin, sectioned, and stained with hematoxylin and eosin. Slides were screened with 5× objectives on a light microscope (Leica DMLB, Leica Microsystems). Analyses were done using ProGres Capture Pro 2.8.8. (Optical Systems, Jenoptick AG).

### Intratracheal administration of *Mtb*-infected pHSCs

For challenge, 8- to 10-week-old female *Rag2*^–/–^*Il2rg*^–/–^mice were anesthetized by i.p. administration of ketamine (50 mg/kg) and Rompun (5 mg/kg). Thereafter, using a micropipette, 100 CFU of *Mtb* H37Rv, 1,000 pHSCs of uninfected and *Mtb*-infected mice at day 28 p.i., or 1,000 Lin^+^ cells and pHSCs of human IGRA^+^ as well as IGRA^−^donors diluted in 50 μl of sterile PBS were gently placed in the trachea of each mouse. Mice were monitored daily for loss of body weight and abdominal respiration. Mice were sacrificed by cervical dislocation 3 weeks post-transfer and lungs were analyzed for *Mtb*-specific DNA, CFUs and histologically.

### Statistics

For all statistical analyses, PRISM (Version 6, GraphPad, San Diego) software was used. Dispersion is presented as the median + interquartile, unless stated otherwise. Statistical analysis was performed with Mann-Whitney two-tailed test. *P* values <0.05 were considered significant.

## Supporting Information

S1 Fig Sorting strategy in human and mouse. (A) Purification of Lin^+^, Lin^–^CD34^+^, Lin^–^CD34^+^CD38^–^CD90^+^, Lin^–^CD34^+^CD38^+^CD90^–^ as well as Lin^–^SP^+^ and Lin^+^ SP^−^cells by FACS from blood cells from IGRA^+^ and IGRA^−^donors. (B) Purification of CD271+CD45- mesenchymal stem cells by FACS from blood cells from IGRA^+^ donors. (C) Purification of Lin^−^hematopoietic progenitors and (D) Lin^+^ Gr1^+^ granulocytes, CD11c^+^ dendritic cells, Mac1^+^ macrophages, NK 1.1^+^ NK cells, CD4^+^/8^+^ T cells and CD19^+^/B220^+^ B cells by FACS from bone marrow of infected mice day 28 p.i. Representative FACS blots are shown. The data contained herein relate to both main Figs 1 and 2.
(DOC)

S2 Fig Quantification of *Mtb*-specific DNA by serial and limiting dilutions of genomic DNA from purified human and mouse pHSCs. (A) Representative example (Donor 14) of a gel analysis of single *Mtb* DNA samples expanded by limiting dilution to a single-target *IS6110* PCR. (B) Representative example (Mouse 2) of a gel analysis of single *Mtb* DNA samples expanded by limiting dilution to a single-target *IS6110* PCR. Note: as expected from Poisson’s distribution not all, but in the analysis of human pHSC, only 5 of 23 individual PCR tests (left), and of mouse LT-pHSC only 6 of 23 individual PCR tests (right) yielded a PCR product (see arrow). The data contained herein relate to both main Figs 1 and 2.
(DOC)

S3 Fig Analysis of qPCR products. (A) Analysis of SYBR green qPCR products by gel electrophoresis, to ensure that qPCR background did not result from a contamination by genomic DNA, thus the amplification of a *Mtb* specific DNA fragment. (B) Amplification plot for the “water control” (1) and for 1 *Mtb* DNA copy (2). (C) Melt curve for the “water control” (1) and for 1 *Mtb* DNA copy (2). The data contained herein relate to both main Figs 1 and 2.
(DOC)

S4 Fig Human peripheral Lin^–^CD34^+^ progenitors as well as SP^+^ pHSCs of IGRA^+^ donors harbour *Mtb* DNA. Genomic DNA was prepared and DNA of 10^3^ hematopoietic progenitors from IGRA^+^ donors was tested by PCR. Quantification of *Mtb*-specific DNA was done by real-time TaqMan PCR using probes that target *MPB64* and *IS6110* together as well as real-time SYBR green PCR using primers that target *MPB64* alone. PCRs were performed in technical triplicates and normalized to human GAPDH (median + interquartile). Due to a lack of sufficient DNA material we were not able to include single-target qPCRs on donors 8 and 9.
(DOC)
