# Supplementary material for: Human and Mouse Hematopoietic Stem Cells Are a Depot for Dormant Mycobacterium tuberculosis
Source: PLoS One. 2017 Jan 3;12(1):e0169119. doi: 10.1371/journal.pone.0169119 (PMC5207496; doi:10.1371/journal.pone.0169119)
Supplement: S1 Fig — (A) Purification of Lin+, Lin–CD34+, Lin–CD34+CD38–CD90+, Lin–CD34+CD38+CD90– as well as Lin–SP+ and Lin+ SP−cells by FACS from blood cells from IGRA+ and IGRA−donors. (B) Purification of CD271+CD45- mesenchymal stem cells by FACS from blood cells from IGRA+ donors. (C) Purification of Lin−hematopoietic progenitors and (D) Lin+ Gr1+ granulocytes, CD11c+ dendritic cells, Mac1+ macrophages, NK 1.1+ NK cells, CD4+/8+ T cells and CD19+/B220+ B cells by FACS from bone marrow of infected mice day 28 p.i. Representative FACS blots are shown. The data contained herein relate to both main Figs 1 and 2. (DOC) [file pone.0169119.s001.doc]

#

**S1 Fig. Sorting strategy in human and mouse.** (A) Purification of Lin^+^, Lin^–^CD34^+^, Lin^–^CD34^+^CD38^–^CD90^+^, Lin^–^CD34^+^CD38^+^CD90^–^ as well as Lin^–^SP^+^ and Lin^+^ SP^–^ cells by FACS from blood cells from IGRA^+^ and IGRA^–^ donors. (B) Purification of CD271^+^CD45^–^ mesenchymal stem cells by FACS from blood cells from IGRA^+^ donors. (C) Purification of Lin^–^ hematopoietic progenitors and (D) Lin^+^ Gr1^+^ granulocytes, CD11c^+^ dendritic cells, Mac1^+^ macrophages, NK 1.1^+^ NK cells, CD4^+^/8^+^ T cells and CD19^+^ B or dendritic cells by FACS from bone marrow of infected mice day 28 p.i. Representative FACS blots are shown. The data contained herein relate to both main Fig 1 and 2.
