# Supplementary material for: Human and Mouse Hematopoietic Stem Cells Are a Depot for Dormant Mycobacterium tuberculosis
Source: PLoS One. 2017 Jan 3;12(1):e0169119. doi: 10.1371/journal.pone.0169119 (PMC5207496; doi:10.1371/journal.pone.0169119)
Supplement: S2 Fig — (A) Representative example (Donor 14) of a gel analysis of single Mtb DNA samples expanded by limiting dilution to a single-target IS6110 PCR. (B) Representative example (Mouse 2) of a gel analysis of single Mtb DNA samples expanded by limiting dilution to a single-target IS6110 PCR. Note: as expected from Poisson’s distribution not all, but in the analysis of human pHSC, only 5 of 23 individual PCR tests (left), and of mouse LT-pHSC only 6 of 23 individual PCR tests (right) yielded a PCR product (see arrow). The data contained herein relate to both main Figs 1 and 2. (DOC) [file pone.0169119.s002.doc]

#

**S2 Fig. Quantification of *Mtb*-specific DNA by serial and limiting dilutions of genomic DNA from purified human and mouse pHSCs.** (A) Representative example (Donor 14) of a gel analysis of single *Mtb* DNA samples expanded by limiting dilution to a single-target *IS6110* PCR. (B) Representative example (Mouse 2) of a gel analysis of single *Mtb* DNA samples expanded by limiting dilution to a single-target *IS6110* PCR. Note: as expected from Poisson’s distribution not all, but in the analysis of human pHSC, only 5 of 23 individual PCR tests (left), and of mouse LT-pHSC only 6 of 23 individual PCR tests (right) yielded a PCR product (see arrow). The data contained herein relate to both main Fig 1 and 2.
