# Supplementary material for: Human and Mouse Hematopoietic Stem Cells Are a Depot for Dormant Mycobacterium tuberculosis
Source: PLoS One. 2017 Jan 3;12(1):e0169119. doi: 10.1371/journal.pone.0169119 (PMC5207496; doi:10.1371/journal.pone.0169119)
Supplement: S3 Fig — (A) Analysis of SYBR green qPCR products by gel electrophoresis, to ensure that qPCR background did not result from a contamination by genomic DNA, thus the amplification of a Mtb specific DNA fragment. (B) Amplification plot for the “water control” (1) and for 1 Mtb DNA copy (2). (C) Melt curve for the “water control” (1) and for 1 Mtb DNA copy (2). The data contained herein relate to both main Figs 1 and 2. (DOC) [file pone.0169119.s003.doc]

#

**Fig S3. Analysis of qPCR products.** (A) Analysis of SYBR green qPCR products by gel electrophoresis, to ensure that qPCR background did not result from a contamination by genomic DNA, thus the amplification of a *Mtb* specific DNA fragment. (B) Amplification plot for the “water control” (1) and for 1 *Mtb* DNA copy (2). (C) Melt curve for the “water control” (1) and for 1 *Mtb* DNA copy (2). The data contained herein relate to both main Fig 1 and 2.
