# Supplementary material for: Human and Mouse Hematopoietic Stem Cells Are a Depot for Dormant Mycobacterium tuberculosis
Source: PLoS One. 2017 Jan 3;12(1):e0169119. doi: 10.1371/journal.pone.0169119 (PMC5207496; doi:10.1371/journal.pone.0169119)
Supplement: S4 Fig — Genomic DNA was prepared and DNA of 103 hematopoietic progenitors from IGRA+ donors was tested by PCR. Quantification of Mtb-specific DNA was done by real-time TaqMan PCR using probes that target MPB64 and IS6110 together as well as real-time SYBR green PCR using primers that target MPB64 alone. PCRs were performed in technical triplicates and normalized to human GAPDH (median + interquartile). Due to a lack of sufficient DNA material we were not able to include single-target qPCRs on donors 8 and 9. (DOC) [file pone.0169119.s004.doc]

| probes/primers | **n *Mtb* DNA copies/ 10^3^ CD34^+^ cells** | | | | | |
| --- | --- | --- | --- | --- | --- | --- |
|  | Donor 10 | Donor 11 | Donor 12 | Donor 13 | Donor 14 | Donor 15 |
| IS6110/MPB64 | 9 ± 5 | 7 ± 4 | 10 ± 5 | 10± 3 | 16 ± 2 | 10 ± 2 |
| MPB64 | 4 ± 2 | 5 ± 3 | 1 ± 0.5 | 2 ± 1 | 7 ± 3 | 7 ± 4 |

**S4 Fig. Human peripheral Lin^–^CD34^+^ progenitors as well as SP^+^ pHSCs of IGRA^+^ donors harbour *Mtb* DNA.** Genomic DNA was prepared and DNA of 10^3^ hematopoietic progenitors from IGRA^+^ donors was tested by PCR. Quantification of *Mtb*-specific DNA was done by real-time TaqMan PCR using probes that target *MPB64* and *IS6110* together as well as real-time SYBR green PCR using primers that target *MPB64* alone. PCRs were performed in technical triplicates and normalized to human GAPDH (median + interquartile). Due to a lack of sufficient DNA material we were not able to include single-target qPCRs on donors 8 and 9.
